# AUNIP Expression Is Correlated With Immune Infiltration and Is a Candidate Diagnostic and Prognostic Biomarker for Hepatocellular Carcinoma and Lung Adenocarcinoma

**DOI:** 10.3389/fonc.2020.590006

**Published:** 2020-12-09

**Authors:** Chenxi Ma, Wenyan Kang, Lu Yu, Zongcheng Yang, Tian Ding

**Affiliations:** Department of Periodontology, School and Hospital of Stomatology, Cheeloo College of Medicine, Shandong University and Shandong Key Laboratory of Oral Tissue Regeneration and Shandong Engineering Laboratory for Dental Materials and Oral Tissue Regeneration, Jinan, China

**Keywords:** AUNIP, tumor-infiltrating, prognosis, hepatocellular carcinoma, lung adenocarcinoma

## Abstract

AUNIP, a novel prognostic biomarker, has been shown to be associated with stromal and immune scores in oral squamous cell carcinoma (OSCC). Nonetheless, its role in other cancer types was unclear. In this study, AUNIP expression was increased in hepatocellular carcinoma (HCC) and lung adenocarcinoma (LUAD) according to data from The Cancer Genome Atlas (TCGA) database, Integrative Molecular Database of Hepatocellular Carcinoma (HCCDB), and Gene Expression Omnibus (GEO) database (GSE45436, GSE102079, GSE10072, GSE31210, and GSE43458). Further, according to copy number variation analysis, AUNIP up-regulation may be associated with copy number variation. Immunohistochemistry showed AUNIP expression was higher in HCC and LUAD compared with the normal tissues. Receiver operating characteristic (ROC) curve analysis demonstrated that AUNIP is a candidate diagnostic biomarker for HCC and LUAD. Next, TCGA, International Cancer Genome Consortium (ICGC), and GEO (GSE31210 and GSE50081) data showed that increased AUNIP expression clearly predicted poor overall survival (OS), disease-specific survival (DSS), and progression-free interval (PFI) in HCC and LUAD. Additionally, multivariate Cox regression analysis involving various clinical factors showed that AUNIP is an independent prognostic biomarker for HCC and LUAD. Next, the role of AUNIP in HCC and LUAD was explored via a co-expression analysis, Gene Ontology (GO) and Kyoto Encyclopedia of Genes and Genomes (KEGG) enrichment analyses, and a gene set variation analysis (GSVA). HCC and LUAD exhibited almost identical enrichment results. More specifically, high AUNIP expression was associated with DNA replication, cell cycle, oocyte meiosis, homologous recombination, mismatch repair, the p53 signal transduction pathway, and progesterone-mediated oocyte maturation. Lastly, the Tumor Immune Estimation Resource (TIMER) tool was used to determine the correlations of AUNIP expression with tumor immune infiltration. AUNIP expression was positively correlated with the infiltration degree of B cells, CD4+ T cells, CD8+ T cells, neutrophils, macrophages, and dendritic cells in HCC. However, AUNIP expression was negatively correlated with the infiltration degree of B cells, CD4+ T cells, and macrophages in LUAD. In addition, AUNIP expression was correlated with immune infiltration in various other tumors. In conclusion, AUNIP, which is associated with tumor immune infiltration, is a candidate diagnostic and prognostic biomarker for HCC and LUAD.

## Introduction

Hepatocellular carcinoma (HCC), a frequently occurring primary liver cancer, is the second most common cause of cancer-related death globally (1, 2), while lung cancer is the leading cause of cancer-related death globally (3). Among lung cancer cases, non-small cell-lung cancer (NSCLC) accounts for 85% of cases, with lung adenocarcinoma (LUAD) being a common histological subtype (4). Despite the tremendous progress regarding treatment options in recent years, the mortality rates of HCC and LUAD are still very high (5, 6). The tumor microenvironment (TME) greatly affects cancer prognosis (7). Immunocytes often infiltrate the TME, and the tumor cells constantly interact with them in this compartment (8). Typically, the infiltration of cytotoxic T cells (CTLs) is related to good prognosis. However, the infiltration of myeloid-derived suppressor cells (MDSCs), tumor-associated macrophages (TAMs), and regulatory T cells (Tregs) is related to poor prognosis (7). Immunotherapy targeting interactions between immune cells and tumor cells has shown promising results in some cancer patients. Some NSCLC patients benefit from treatment based on Treg blockade (9), while antiangiogenics and immune checkpoint inhibitors can be effective for HCC (2). However, only a sed proportion of cancer patients achieve a good treatment effect (10). Therefore, identifying further prognostic biomarkers and possible therapeutic targets is of great significance.

AUNIP (Aurora kinase A [Aurora-A] and ninein-interacting protein), also known as AIBp and C1orf135, is a centrosome protein that promotes the maintenance of the centrosome structure and the formation of the spindle by interacting with Aurora-A and Ninein (11). AUNIP can control Plk1 and Aurora-A activation, thereby modulating mitotic entry and mitotic spindle assembly (12). Moreover, high AUNIP expression has been detected in brain tumors (11). Furthermore, recent research has found that AUNIP regulates the cell cycle of oral squamous cell carcinoma (OSCC) cells and can be used as a prognostic biomarker for OSCC. Additionally, AUNIP expression is negatively correlated with stromal and immune scores in OSCC, indicating that AUNIP may affect immune infiltration in OSCC (13). However, the role and mechanism of AUNIP in other types of cancer remain unclear.

In this study, datasets obtained from the International Cancer Genome Consortium (ICGC), Integrative Molecular Database of Hepatocellular Carcinoma (HCCDB), The Cancer Genome Atlas (TCGA), and Gene Expression Omnibus (GEO) databases were utilized for comprehensive analysis of AUNIP expression in different tumor types (HCC and LUAD), including the association of AUNIP expression with prognosis. We analyzed the expression of AUNIP in HCC and LUAD by immunohistochemistry staining. Receiver operating characteristic (ROC) curve analyses were used to evaluate the value of AUNIP as a diagnostic biomarker for HCC and LUAD. Co-expression analysis, Gene Ontology (GO) and Kyoto Encyclopedia of Genes and Genomes (KEGG) enrichment analyses, and gene set variation analysis (GSVA) were used to investigate the role of AUNIP in HCC and LUAD. The Tumor Immune Estimation Resource (TIMER) tool was utilized to determine the potential associations of AUNIP expression with tumor-infiltrating immunocytes. Our findings indicate the diagnostic and prognostic value of AUNIP in HCC and LUAD and demonstrate the potential associations between AUNIP expression and immune infiltration. The mechanism of AUNIP in HCC and LUAD was also investigated.

## Materials and Methods

### Data Acquisition and Preprocessing

Gene expression and survival data for 33 cancer types were acquired from the TCGA module of the Xena Public Data Hubs in the UCSC Xena platform (http://xena.ucsc.edu/) (14). The gene expression levels had been quantified using Illumina HiSeq and normalized using log2(fpkm+1). Additionally, clinical data on HCC and LUAD cases were obtained from the TCGA database (https://cancergenome.nih.gov/). From the Gene Expression Omnibus (GEO) database (http://www.ncbi.nlm.nih.gov/geo/), we selected two HCC datasets GSE45436 (15) and GSE102079 (16) and four LUAD datasets GSE10072 (17), GSE31210 (18), GSE43458 (19), and GSE50081 (20) for bioinformatics analysis (involving analyzing AUNIP expression in cancer tissues relative to non-carcinoma tissues, survival analysis, and analyzing the diagnostic performance of AUNIP). Raw CEL files and platform files from GEO were downloaded. The robust multi-array average (RMA) algorithm was used to process and normalize the raw CEL files of each cohort independently using the “affy” R package (version 1.64.0) (21). In addition, mRNA expression and clinical data (including survival data) on HCC cases were downloaded from the International Cancer Genome Consortium (ICGC) database (https://dcc.icgc.org/releases/current/Projects/LIRI-JP). Lastly, mRNA expression (z-scores) and AUNIP copy number data for HCC and LUAD were downloaded from cBioPortal (https://www.cbioportal.org/) (22, 23).

### Clinical Specimens

Tissue microarray chips were purchased from Shanghai Qutdo Biotech Company (Shanghai, China). Tumor and adjacent normal tissues were collected from 75 patients with HCC and 75 patients with LUAD in Taizhou Hospital (Zhejiang, China). Written informed consent was obtained from all the patients. This study was approved by Taizhou Hospital Ethics Committee (Zhejiang, China).

### HCCDB Data Analysis

The HCCDB database (http://lifeome.net/database/hccdb) (24) contains 15 public datasets that include data on AUNIP mRNA expression in HCC. High AUNIP expression in HCC relative to adjacent non-carcinoma tissues was confirmed using the HCCDB data.

### Co-Expression Analysis

The genes co-expressed with AUNIP were identified in HCC and LUAD using the “limma” R package (version 3.42.2) (25) based on the following thresholds: |correlation coefficient| >0.6 and P-value <0.05. Thereafter, heatmaps of the top 20 genes with positive or negative correlation with AUNIP expression in HCC and LUAD were plotted using the “pheatmap” R package (version 1.0.12).

### GO and KEGG Enrichment Analyses

The genes that were co-expressed with AUNIP in HCC and LUAD were subjected to GO and KEGG enrichment analyses using the “clusterprofiler” R package (version 3.14.3) (26). Q-value <0.05 indicated significant GO terms and KEGG pathways.

### Gene Set Variation Analysis

To validate the top 10 enriched KEGG pathways in HCC and LUAD, the “GSVA” R package (version 1.34.0) (27) was used to calculate normalized enrichment scores (NESs). The control gene set was “c2.cp.kegg.v7.1.symbols.gmt” from the Molecular Signature Database (MSigDB, https://www.gsea-msigdb.org/gsea/downloads.jsp). As some KEGG pathways were not present in the gene set file, NESs could only be calculated for 7 of the top 10 KEGG pathways. The HCC and LUAD cases were divided based on the corresponding median AUNIP expression level, and heatmaps were then constructed using the “pheatmap” R package (version 1.0.12) to visualize the NESs of the KEGG pathways in the pairs of groups.

### Comprehensive Analysis of Tumor-Infiltrating Immune Cells

The TIMER web server (https://cistrome.shinyapps.io/timer/) (28) was developed to comprehensively and systematically analyze immune infiltration in different cancer types. The TIMER algorithm was applied to determine the infiltration degrees of six types of immune cells (CD4+ T cells, CD8+ T cells, B cells, macrophages, neutrophils, and dendritic cells [DCs]), i.e., the estimated immune cell abundances, in 32 cancer types. A heatmap was constructed using the “pheatmap” R package (version 1.0.12) to visualize the immune infiltration degrees in HCC and LUAD. Spearman correlations regarding the immune cell abundances were visualized using Cytoscape (version 3.6.1, https://cytoscape.org/). Next, Spearman correlations between AUNIP expression and the abundances of the six kinds of immune cells among the 32 cancer types were assessed, using the gene module in TIMER. Lastly, Spearman correlation analysis was further employed to examine the correlations between AUNIP expression and the markers of B cells, monocytes, neutrophils, CD8+ T cells, natural killer cells, macrophages, Treg cells, Th1 cells, and DCs in HCC and LUAD, using the TIMER2.0 website (http://timer.cistrome.org/) (29) for visualization; in addition, the analysis was then adjusted for tumor purity.

### Immunohistochemistry Staining

The tissue microarray chips were dewaxed and rehydrated, and then incubated with monoclonal rabbit anti-human AUNIP (dilution 1:200, Bioss, bs-15019R) overnight at 4 °C after epitope retrieval, H2O2 treatment and non-specific antigens blocking. Finally, chips were incubated with secondary antibody and we used DAB staining kit (Vector Laboratories, USA) for signals detection.

### Statistical Analysis

The Wilcoxon rank-sum test was used to compare differences between pairs of groups. Spearman correlation analysis was used to assess the significance of correlations. Kaplan–Meier survival analyses were conducted based on the best separation cut-off value of AUNIP expression using the “survival” R package (version 3.1-11) and the “survminer” R package (version 0.4.6), with differences being assessed using the log-rank test. While TCGA, GEO, and ICGC data were used to assess the relationship between high AUNIP expression and overall survival (OS), TCGA data were also used to assess the relationship between high AUNIP expression and both disease-specific survival (DSS; defined as the duration between diagnosis and disease-related death) and progression-free interval [PFI; defined as the duration between diagnosis and the onset of a new tumor event, including local relapse, disease progression, new primary tumor, distant metastasis, or cancer-related death (30)]. Furthermore, univariate and multivariate Cox regression analyses were utilized to determine whether AUNIP expression might serve as a prognostic factor independent of other clinical variables, using TCGA data. ROC curves were generated using MedCalc software. R software (version 3.6.3, https://www.r-project.org/) was used for the other statistical analyses. P<0.05 was deemed to be statistically significant.

## Results

### AUNIP Expression in 33 Cancer Types

**Figure 1:**
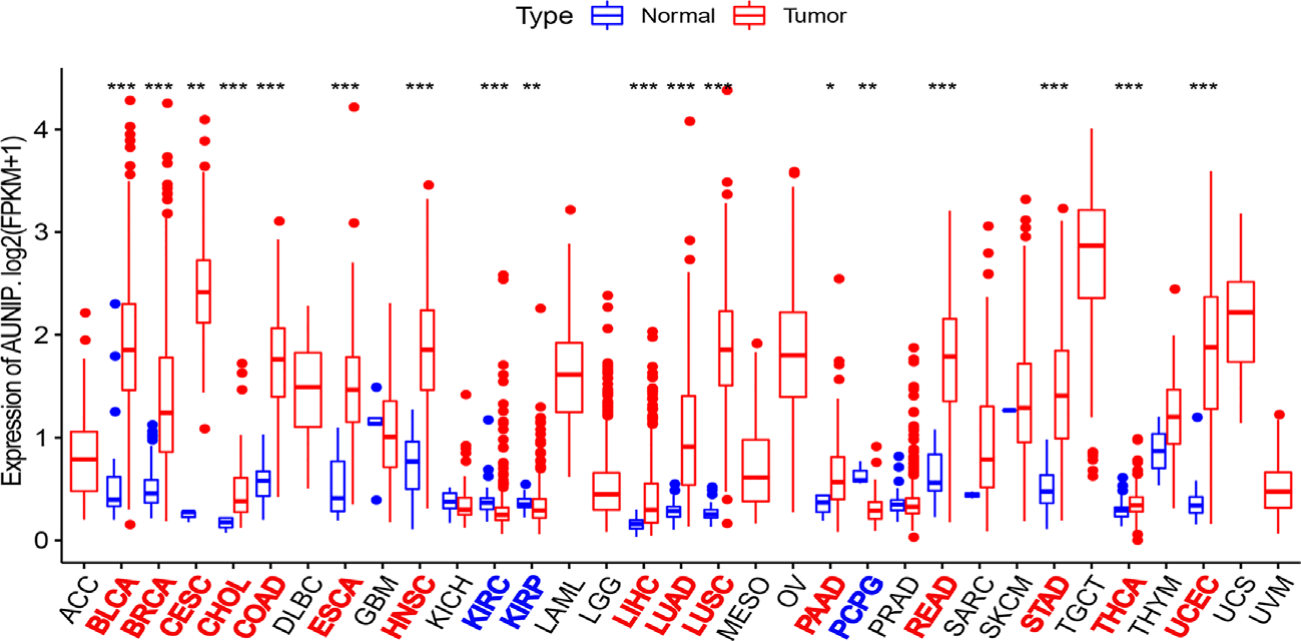
AUNIP expression in 33 cancer types based on TCGA data. *P < 0.05, **P < 0.01, ***P < 0.001.

RNA-seq data from the TCGA database were used to compare AUNIP expression between tumor and normal tissues. AUNIP expression was markedly and significantly increased in LUAD, liver hepatocellular carcinoma (LIHC), colon adenocarcinoma (COAD), breast invasive carcinoma (BRCA), cervical squamous cell carcinoma and endocervical adenocarcinoma (CESC), cholangiocarcinoma (CHOL), bladder urothelial carcinoma (BLCA), head and neck squamous cell carcinoma (HNSC), esophageal carcinoma (ESCA), pancreatic adenocarcinoma (PAAD), lung squamous cell carcinoma (LUSC), rectum adenocarcinoma (READ), stomach adenocarcinoma (STAD), uterine corpus endometrial carcinoma (UCEC), and thyroid carcinoma (THCA), relative to that in corresponding non-carcinoma tissues (**Figure 1**). However, AUNIP expression in pheochromocytoma and paraganglioma (PCPG), kidney renal papillary cell carcinoma (KIRP), and kidney renal clear cell carcinoma (KIRC) significantly decreased relative to that in corresponding non-carcinoma tissues.

### High AUNIP Expression Was Related to Poor Prognosis for Various Tumors

**Figure 2:**
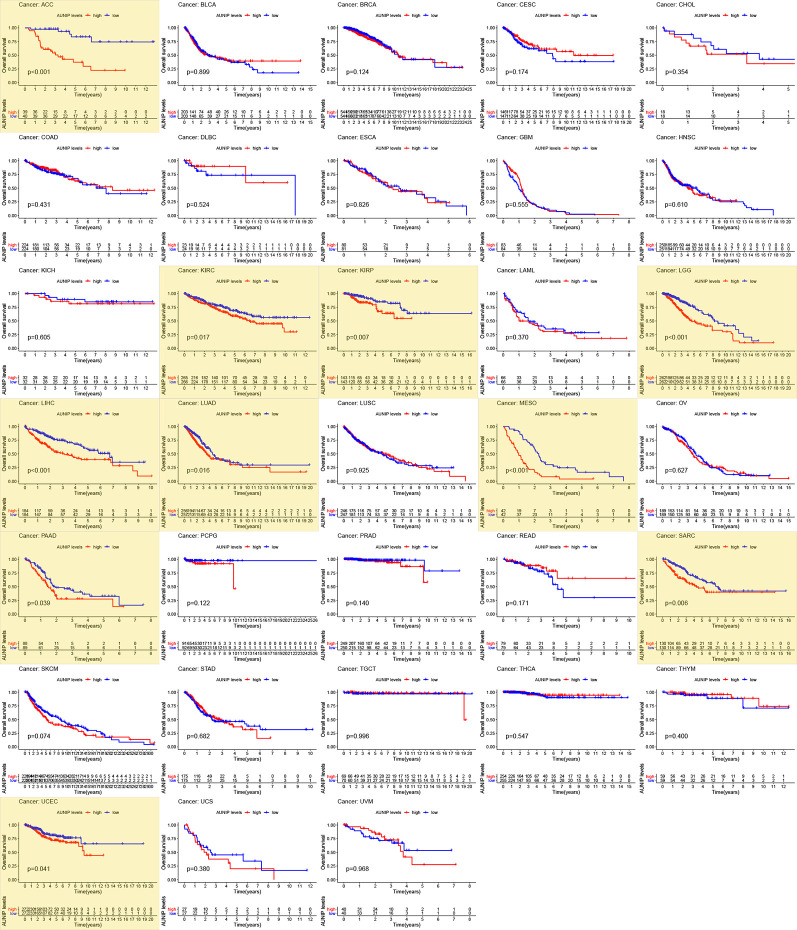
Survival analysis of 33 cancer types based on TCGA data. P < 0.05 indicates statistical significance and the significant values are marked in yellow.

Next, RNA-seq and survival data from the TCGA database were used to investigate whether AUNIP expression was associated with OS in a pan-cancer analysis (**Figure 2**). AUNIP expression was associated with OS in 10 diverse types of cancers, namely, LIHC, LUAD, PAAD, UCEC, KIRC, KIRP, sarcoma (SARC), adrenocortical carcinoma (ACC), brain lower grade glioma (LGG), and mesothelioma (MESO). Among these tumors, AUNIP expression increased in four types (LIHC, LUAD, PAAD, and UCEC) relative to adjacent non-carcinoma tissues, and for a further three (ACC, LGG and MESO), we could not determine whether AUNIP expression increased in the cancer tissues relative to adjacent non-carcinoma tissues due to lack of data on adjacent non-carcinoma tissues. Finally, we selected HCC/LIHC and LUAD to carry out further analyses based on the size of the tumor sample datasets.

### AUNIP Expression Was High in HCC and LUAD

**Figure 3:**
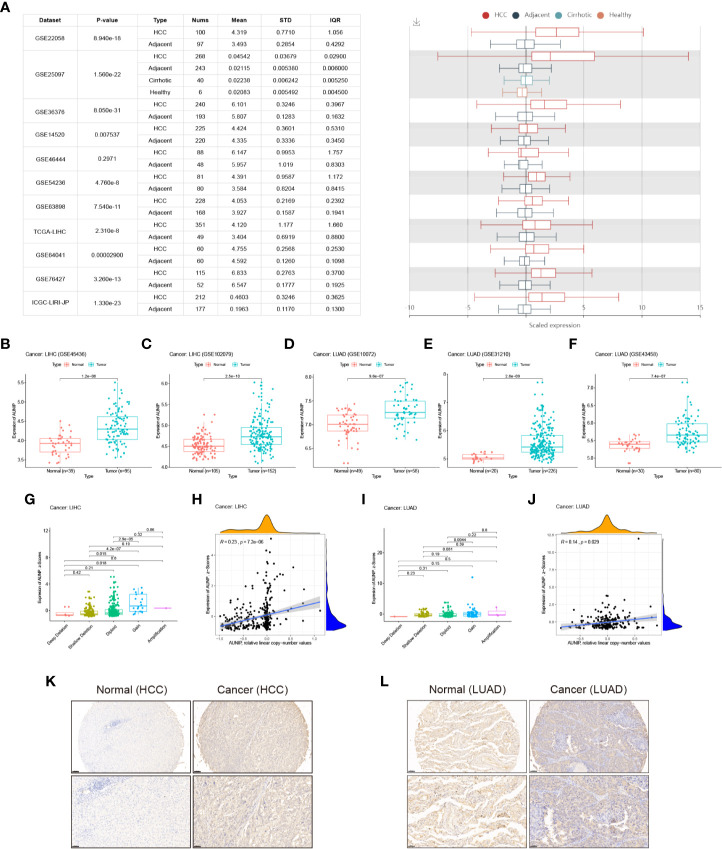
AUNIP expression in HCC and LUAD. **(A)** Chart and plot of AUNIP expression in HCC and matched non-carcinoma tissues based on HCCDB data. Box plots of AUNIP expression in HCC and adjacent normal tissues in the **(B)** GSE45436 and **(C)** GSE102079 cohorts. Box plots of AUNIP expression in LUAD and adjacent normal tissues in the **(D)** GSE10072, **(E)** GSE31210, and **(F)** GSE43458 cohorts. Dot plot and correlation graph showing the positive correlation between AUNIP expression (z-scores) and AUNIP copy number values in **(G, H)** HCC and **(I, J)** LUAD. **(K)** Representative IHC staining for AUNIP in HCC and normal tissues. Scale bars: 100 μm (insets 50 μm). (**L**) Representative IHC staining for AUNIP in LUAD and normal tissues. Scale bars: 100 μm (insets 50 μm).

An analysis of 11 HCC cohorts in the HCCDB database showed that AUNIP mRNA expression remarkably increased in HCC relative to adjacent non-carcinoma tissues in 10 HCC cohorts (**Figure 3A**), with the difference being non-significant in the remaining HCC cohort. Moreover, an analysis of two HCC cohorts in the GEO database (GSE45436 and GSE102079) showed that AUNIP was highly expressed in HCC in both cohorts (**Figures 3B, C**), while an analysis of three LUAD cohorts in the GEO database (GSE10072, GSE31210, and GSE43458) showed that AUNIP was highly expressed in LUAD in all three cohorts (**Figures 3D–F**). These results strongly indicate the up-regulation of AUNIP in HCC and LUAD. Next, as copy number amplification is a genetic mechanism underlying oncogene up-regulation, we analyzed AUNIP mRNA expression (z-scores) and copy number data regarding HCC and LUAD from cBioPortal. As expected, AUNIP expression increased significantly in the Gain group and was significantly correlated with copy number amplification in both HCC and LUAD (**Figures 3G–J**). Therefore, copy number variation may be a primary mechanism underlying AUNIP up-regulation in HCC and LUAD. Finally, to validate the protein levels of AUNIP in HCC and LUAD, we performed immunohistochemistry and found that the expression of AUNIP was elevated in HCC (**Figure 3K**) and LUAD (**Figure 3L**) compared with adjacent normal tissues.

### Multifaceted Prognostic Value of AUNIP in HCC and LUAD

**Figure 4:**
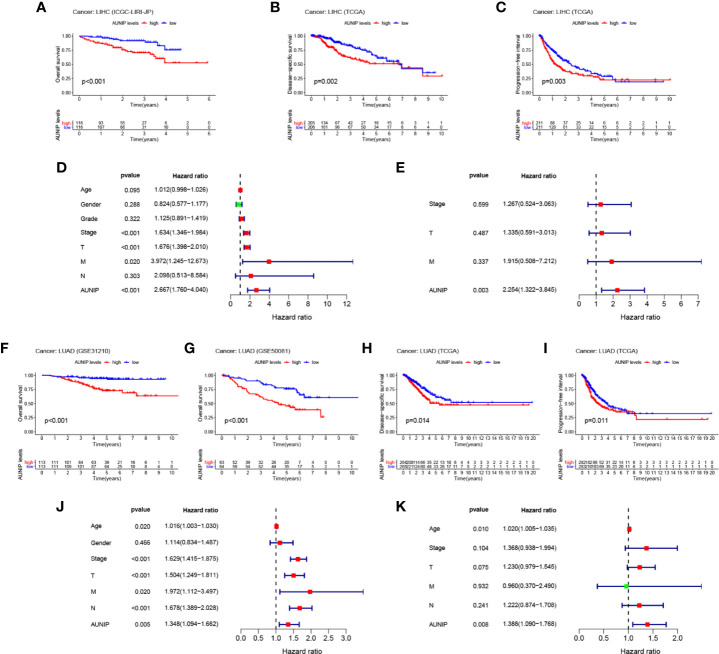
Multifaceted prognostic value of AUNIP in HCC/LIHC and LUAD. **(A)** Overall survival (OS) of ICGC liver cancer, RIKEN, Japan (LIRI-JP) cases. **(B)** Disease-specific survival (DSS) of TCGA LIHC cases. **(C)** Progression-free interval (PFI) of TCGA LIHC cases. **(D)** Univariate and **(E)** multivariate Cox regression analyses of OS-related factors among TCGA LIHC cases. **(F)** OS of GSE31210 LUAD cases. **(G)** OS of GSE50081 LUAD cases. **(H)** DSS of TCGA LUAD cases. **(I)** PFI of TCGA LUAD cases. **(J)** Univariate and **(K)** multivariate Cox regression analyses of OS-related factors among TCGA LUAD cases.

To verify that high AUNIP expression was related to poor survival in HCC and LUAD, we analyzed ICGC and GEO data. We found that the low AUNIP expression group had better OS for both the HCC cases (ICGC: P<0.001) and the LUAD cases (GSE31210: P<0.001; GSE50081: P<0.001) (**Figures 4A, F, G**). Furthermore, the low AUNIP expression group also had better DSS (HCC: P=0.002; LUAD: P=0.014) and PFI (HCC: P=0.003; LUAD: P=0.011) for both the HCC and LUAD cases in the TCGA datasets (**Figures 4B, C, H, I**). Finally, univariate and multivariate Cox regression analyses of TCGA data were used to evaluate whether AUNIP expression is a prognostic factor in HCC and LUAD. The univariate results indicated that AUNIP expression might serve as a factor for predicting OS in HCC and LUAD (**Figures 4D, J**), which was verified by the multivariate results, which indicated the potential of AUNIP expression as an independent predictor of OS in HCC and LUAD (**Figures 4E, K**). ROC curve analysis of 1-, 3-, and 5-year OS were conducted using TCGA data. Regarding HCC, the area under the curve (AUC) values were 0.662, 0.635, and 0.586, respectively. Regarding LUAD, the AUC values were 0.572, 0.580, and 0.549, respectively (**Supplementary Figure S1**).

### Diagnostic Performance of AUNIP in HCC and LUAD

**Figure 5:**
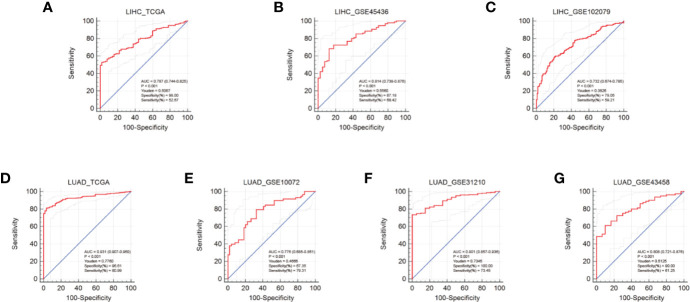
Diagnostic performance of AUNIP in HCC and LUAD. ROC curves evaluating the diagnostic performance of AUNIP for HCC patients in the **(A)** TCGA LIHC, **(B)** GSE45436, and **(C)** GSE102079 cohorts. ROC curves evaluating the diagnostic performance of AUNIP for LUAD patients in the **(D)** TCGA LUAD, **(E)** GSE10072, **(F)** GSE31210, and **(G)** GSE43458 cohorts.

To evaluate the diagnostic performance of AUNIP in HCC and LUAD, we conducted ROC curve analyses. Regarding HCC, the mean AUC values were 0.787 (TCGA), 0.814 (GSE45436), and 0.732 (GSE102079), respectively (**Figures 5A–C**). Regarding LUAD, the mean AUC values were 0.931 (TCGA), 0.776 (GSE10072), 0.901 (GSE31210), and 0.808 (GSE43458), respectively (**Figures 5D–G**). These results indicate that AUNIP had good diagnostic performance in HCC and LUAD.

### Functional Enrichment Analysis of Genes That Were Co-Expressed With AUNIP

**Figure 6:**
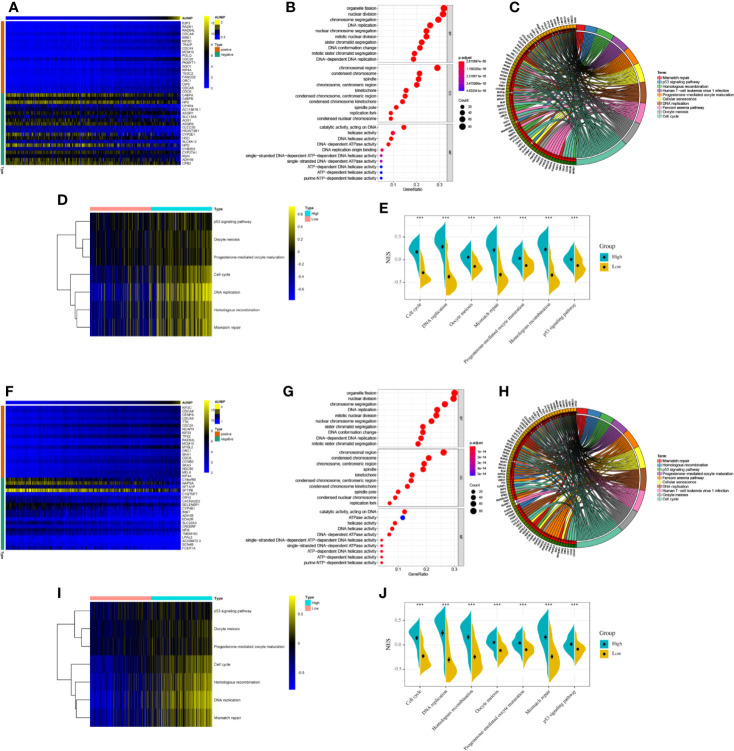
Functional enrichment analyses of AUNIP in HCC and LUAD. **(A)** Heatmap of 20 genes with the most significant correlations with AUNIP in HCC. **(B)** GO and **(C)** KEGG analysis of the co-expressed genes in HCC. **(D)** Heatmap and **(E)** violin plot of the normalized enrichment scores (NESs) for seven pathways between high and low AUNIP expression groups for HCC. **(F)** Heatmap of 20 genes with the most significant correlations with AUNIP in LUAD. **(G)** GO and **(H)** KEGG analysis of the co-expressed genes in LUAD. **(I)** Heatmap and **(J)** violin plot of the NESs for seven pathways between high and low AUNIP expression groups for LUAD. *P < 0.05, **P < 0.01, ***P < 0.001.

To explore the biological function of AUNIP in HCC and LUAD, we conducted a co-expression analysis of AUNIP. Heatmaps of the top 20 genes positively and negatively associated with AUNIP in HCC and LUAD were plotted (**Figures 6A, F**). The thresholds for co-expression were |correlation coefficient|>0.6 and p<0.05. Next, co-expression genes were chosen to perform GO and KEGG analyses. In HCC, we detected enrichment in several biological process GO terms such as organelle fission, nuclear division, chromosome segregation, DNA replication, nuclear chromosome segregation, mitotic nuclear division, sister chromatid segregation, DNA conformation change, mitotic sister chromatid segregation, and DNA-dependent DNA replication (**Figure 6B**). In terms of cellular components, the chromosomal region was the most significantly enriched GO term. Furthermore, some molecular component GO terms, such as catalytic activity, acting on DNA, helicase activity, DNA helicase activity, DNA-dependent ATPase activity, DNA replication origin binding, single-stranded DNA-dependent ATP-dependent DNA helicase activity, single-stranded DNA-dependent ATPase activity, ATP-dependent DNA helicase activity, ATP-dependent helicase activity, and purine NTP-dependent helicase activity, were enriched. As for KEGG pathway analysis, genes associated with the cell cycle, DNA replication, the Fanconi anemia pathway, homologous recombination, oocyte meiosis, progesterone-mediated oocyte maturation, cellular senescence, mismatch repair, the p53 signaling pathway, and human T-cell leukemia virus 1 infection, were mostly associated with AUNIP expression (**Figure 6C**). To our surprise, our GO and KEGG pathway enrichment analyses results were almost identical for HCC and LUAD. This indicated that the mechanism of AUNIP activity might be the same in both HCC and LUAD (**Figures 6G, H**). Meanwhile, GSVA confirmed that the NESs for 7 of these 10 pathways in HCC and LUAD (i.e., cell cycle, DNA replication, homologous recombination, oocyte meiosis, progesterone-mediated oocyte maturation, mismatch repair, and the p53 signaling pathway) were significantly increased in the high expression group of AUNIP (**Figures 6D, E, I, J**).

### AUNIP Expression Was Related to Immune Infiltration in HCC and LUAD

**Figure 7:**
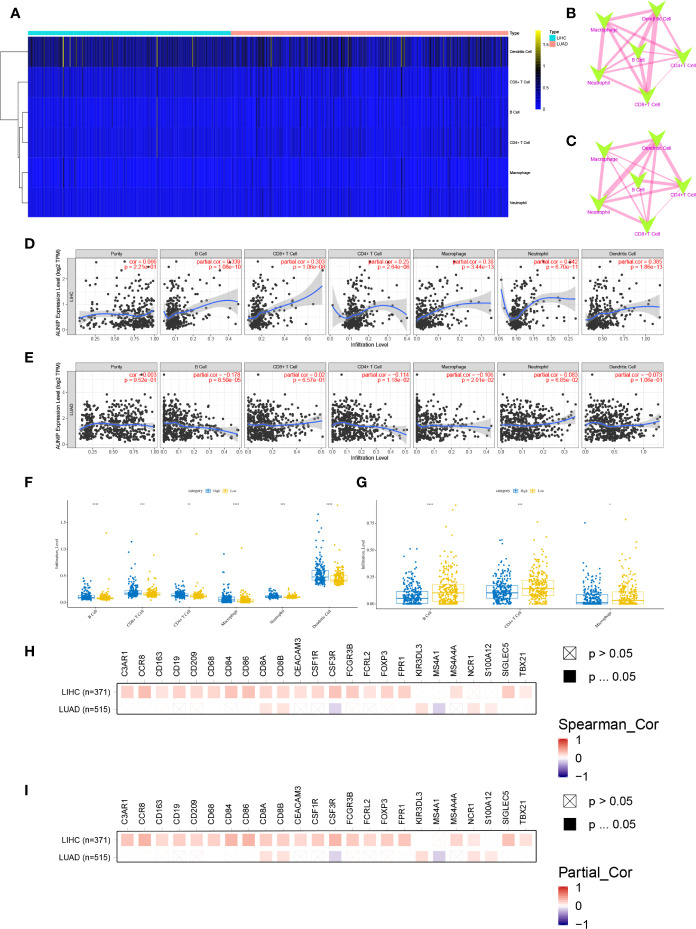
AUNIP expression is related to the immune infiltration degrees in HCC and LUAD. **(A)** Heatmap of the abundances of six infiltrating immune cell types in HCC and LUAD. Spearman correlations between immune cell abundances in **(B)** HCC and **(C)** LUAD. Blue and red represent negative and positive correlation, respectively, and the thicker the line, the larger the correlation coefficient. Spearman correlations between AUNIP expression and the immune cell abundances in **(D)** HCC and **(E)** LUAD. Comparisons of immune cell abundances between the high and low AUNIP expression groups in **(F)** HCC and **(G)** LUAD. Spearman correlations between AUNIP expression and markers of B cells, monocytes, neutrophils, CD8+ T cells, natural killer cells, macrophages, Treg cells, Th1 cells, and DCs in HCC and LUAD **(H)** before and **(I)** after adjusting for tumor purity. *P < 0.05, **P < 0.01, ***P < 0.001.

Using TIMER, the infiltration degrees of six cell types (B cells, CD4+ T cells, CD8+ T cells, neutrophils, macrophages, and DCs) were estimated. DCs were the most abundant of these cell types in the TME in HCC and LUAD (**Figure 7A**). Although the correlations between the infiltration abundances of the six immune cells were not exactly the same for HCC and LUAD, the correlations were all positive (**Figures 7B, C**). Next, the correlations between AUNIP expression and immune cell infiltration in HCC and LUAD were assessed. As a result, AUNIP expression was found to be positively associated with the infiltration degrees of B cells (r=0.339, P=1.08e-10), CD4+ T cells (r=0.25, P=2.64e-06), CD8+ T cells (r=0.303, P=1.06e-08), neutrophils (r=0.342, P=6.70e-11), macrophages (r=0.38, P=3.44e-13), and DCs (r=0.385, P=1.86e-13) in HCC (**Figure 7D**). Similarly, there were significant differences in the infiltration degrees of these cells between the high and low AUNIP expression groups for HCC (**Figure 7F**). However, regarding LUAD, AUNIP expression was negatively correlated with the infiltration degrees of B cells (r = −0.178, P=8.50e-05), macrophages (r = −0.106, P=2.01e-02), and CD4+ T cells (r = −0.114, P=1.18e-02) (**Figure 7E**), and there were significant differences in the infiltration degrees of these cells between high and low AUNIP expression groups for LUAD (**Figure 7G**). We then further explored the correlations between AUNIP expression and genetic markers of various immune cell types (B cells, CD8+ T cells, neutrophils, macrophages, DCs, natural killer cells, Th1 cells, Treg cells, and monocytes). As a result, AUNIP expression was found to be significantly correlated with 20 and 7 markers in HCC and LUAD, respectively (**Figure 7H** and **Supplementary Table S1**), and 21 and 7 markers, respectively, after correction for tumor purity (**Figure 7I** and **Supplementary Table S2**). These results further demonstrated that AUNIP expression is related to immune cell infiltration degrees in HCC and LUAD.

### AUNIP Expression Was Associated With Immune Infiltration in Additional Cancer Types

**Figure 8:**
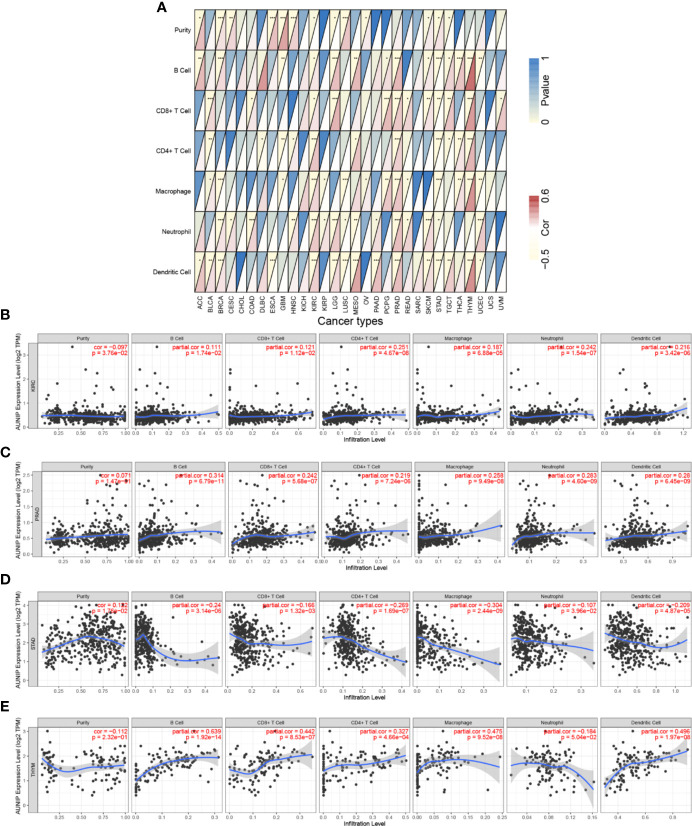
AUNIP expression is associated with immune infiltration degrees in additional cancer types. Spearman correlations between AUNIP expression and six infiltrating immunocyte abundances in **(A)** 30 cancer types, **(B)** KIRC, **(C)** PRAD, **(D)** STAD, and **(E)** THYM. *P < 0.05, **P < 0.01, ***P < 0.001.

We also evaluated the correlations between AUNIP expression and immune infiltration in other cancer types. AUNIP expression was significantly related to tumor purity in 13 cancer types and to the B cell infiltration degree in 14 cancer types (**Figure 8A** and **Supplementary Table S3**). In addition, AUNIP expression was significantly related to the infiltration degree of CD4+ T cells, CD8+ T cells, neutrophils, macrophages, and DCs in 11, 12, 15, 14, and 15 cancer types, respectively. Furthermore, AUNIP expression was markedly correlated with the infiltration degrees of at least four immunocyte types in each of four cancer types, comprising KIRC, STAD, prostate adenocarcinoma (PRAD), and thymoma (THYM) (**Figures 8B–E**).

## Discussion

AUNIP (Aurora kinase A and ninein-interacting protein) is responsible for maintaining the centrosomal structure and facilitating spindle formation (11). AUNIP has not been thoroughly researched, but it is a potential prognostic biomarker for OSCC. In addition, AUNIP is related to immune and stromal scores in OSCC, which suggests that it may be involved in recruiting infiltrating immune and stromal cells to the TME in OSCC (13). We detected changes in AUNIP mRNA expression based on a pan-cancer analysis. Additionally, we explored the associations of AUNIP expression with both prognosis and immune infiltration. We conducted further analysis on HCC and LUAD, as they were associated with more cancer-related deaths, exploring not only the associations of AUNIP expression with prognosis and immune infiltration, but also the diagnostic performance of AUNIP.

TCGA data were utilized to analyze the AUNIP mRNA expression in diverse cancer types. As a result, the AUNIP mRNA expression was found to be markedly increased in most cancers, comprising LUAD, LIHC, COAD, BRCA, BLCA, CHOL, CESC, STAD, ESCA, THCA, READ, LUSC, HNSC, PAAD, and UCEC (**Figure 1**). However, AUNIP was down-regulated in other cancer types, comprising KIRC, KIRP, and PCPG, which might be due to differences in the underlying pathogenic mechanisms involved in these cancers. In addition, by analyzing multiple datasets from different databases, we fully verified the high AUNIP expression in HCC and LUAD compared to non-carcinoma tissues (**Figures 3A–F**). This was consistent with our immunohistochemistry results (**Figures 3K–L**). To explore the potential reasons for the increased AUNIP expression in HCC and LUAD, we then analyzed the AUNIP gene expression (z-scores) and copy number data for HCC and LUAD from cBioPortal. As the results showed, AUNIP expression significantly increased in the Gain group and AUNIP expression was significantly correlated with copy number amplification in HCC and LUAD (**Figures 3G–J**). This suggested that copy number amplification might be a mechanism underlying AUNIP up-regulation in HCC and LUAD.

AUNIP is a potential diagnostic and prognostic biomarker in OSCC, but its significance as a diagnostic and prognostic biomarker in other cancers has not previously been evaluated. TCGA data indicated that high AUNIP expression was related to poorer OS in 10 diverse cancer types, comprising LUAD, LIHC, ACC, KIRC, KIRP, LGG, MESO, PAAD, SARC, and UCEC (**Figure 2**). In addition, also based on TCGA data, lower AUNIP expression led to better DSS and PFI in HCC and LUAD patients (**Figures 4B, C, H, I**). According to univariate Cox analysis of TCGA data, AUNIP expression was a prognostic factor in HCC and LUAD (**Figures 4D, J**). Subsequent multivariate Cox analysis showed that AUNIP expression was an independent predictor of OS in HCC and LUAD (**Figures 4E, K**). Moreover, GEO and ICGC data confirmed the association of high AUNIP expression with poor OS for HCC and LUAD (**Figures 4A, F, G**). Furthermore, the ROC curve analysis demonstrated that AUNIP expression had good diagnostic performance in HCC and LUAD (**Figure 5**). Overall, these findings provide strong support for the use of AUNIP expression as a diagnostic and prognostic biomarker for HCC and LUAD.

Several potential mechanisms regarding the relationship between high AUNIP expression and poor cancer prognosis have been proposed in recent studies. Cancer is characterized by aberrant cell cycle dynamics that result in uncontrolled proliferation of tumor cells. Aberrant expression of proteins associated with the cell cycle can lead to tumor invasion, metastasis, induce drug resistance, and resist apoptosis. Many cell cycle-related proteins have become cancer therapeutic targets (31). Aurora-A belongs to the Aurora serine/threonine kinase family. It is related to several events in cell cycle transition and is important for bipolar spindle assembly and both mitosis and meiosis (32). Aurora-A up-regulation may lead to the development of malignant tumors and may be linked with poor cancer prognosis (33). It has become a priority therapeutic target for treating cancer (34). AUNIP interacts with the C-terminus of Aurora-A, which is necessary for Aurora-A to move dynamically at centrosomes and the spindle apparatus during the cell cycle (11). Based on our enrichment analysis, AUNIP was associated with the cell cycle (**Figures 6C, H**). The association of AUNIP with Aurora-A might underlie the relationship between AUNIP expression and poor cancer prognosis.

DNA double-strand breaks (DSB) represent the worst type of DNA damage. Without prompt and accurate repair, DSB can lead to mutation, genome instability, apoptosis, and even cancer (35). Homologous recombination is a vital DSB repair mechanism. Impairments regarding the homologous recombination-related genes can reduce DSB repair and significantly increase the incidence of tumors (36). Our enrichment analysis results (**Figures 6C, H**) are consistent with research showing that AUNIP directs DSB towards the homologous recombination repair pathway (37). Thus, AUNIP may be involved in DSB repair in HCC and LUAD. Increased DSB repair capabilities can lead to radio- and chemoresistance and, ultimately, cancer recurrence (38). Therefore, AUNIP may serve as a therapeutic target in HCC and LUAD, and more studies are needed for validation.

Further, TIMER was used to explore the correlation of AUNIP expression with immune cell infiltration degrees in tumors, which suggested that AUNIP expression was positively correlated with the infiltration degree of B cells, neutrophils, CD4+ T cells, CD8+ T cells, macrophages, and DCs in HCC (**Figures 7D, F**). However, AUNIP expression was negatively correlated with the infiltration degree of macrophages, B cells, and CD4+ T cells in LUAD (**Figures 7E, G**). Therefore, different infiltrating immunocytes are recruited by different tumors to the TME. Interestingly, AUNIP expression in HCC and LUAD was not associated with tumor purity, which may be due to the equal AUNIP expression in cancer cells and the TME. Furthermore, the correlations of AUNIP expression with genetic markers of various immune cells were explored after adjusting for tumor purity. As a result, we found that AUNIP expression was significantly correlated with 21 and 7 immune cell markers in HCC and LUAD, respectively (**Figure 7I** and **Supplementary Table S2**). In general, the relationships of AUNIP expression with immune cell markers revealed that AUNIP was involved in regulating tumor immunity in HCC and LUAD. In addition, AUNIP was related to the immune infiltration degrees in additional cancer types, including KIRC, PRAD, STAD, and THYM (**Figures 8A–E** and **Supplementary Table S3**). These findings provide strong evidence that AUNIP is involved in tumor immune infiltration. Immunotherapy has transformed the treatment of many advanced malignant tumors (39). The immune responses at tumor sites are determined by the TME-infiltrating immune cells (40). AUNIP may have an important role in recruiting infiltrating immune cells and regulating immunity in HCC and LUAD, thus affecting prognosis.

In conclusion, our findings indicate that AUNIP may serve as a diagnostic and prognostic biomarker for HCC and LUAD. Additionally, we identified pathways associated with AUNIP in HCC and LUAD, such as DNA replication, cell cycle, oocyte meiosis, mismatch repair, homologous recombination, progesterone-mediated oocyte maturation, and the p53 signal transduction pathway. Furthermore, AUNIP expression is related to the tumor infiltration degrees of various immune cells. This study provides a reference for further exploring new immune-based therapies for cancers.

## Data Availability Statement

The datasets presented in this study can be found in online repositories. The names of the repository/repositories and accession number(s) can be found in the article/**Supplementary Material**.

## Ethics Statement

The studies involving human participants were reviewed and approved by Taizhou Hospital Ethics Committee (Zhejiang, China). The patients/participants provided their written informed consent to participate in this study. Written informed consent was obtained from the individual(s) for the publication of any potentially identifiable images or data included in this article.

## Author Contributions

CM designed the study, analyzed the data, performed the immunohistochemistry, and wrote the paper. CM, WK, LY, ZY, and TD were responsible for data extraction from the databases. All authors contributed to the article and approved the submitted version.

## Funding

This study was funded by the National Natural Science Foundation of China (no. 81670993 and 81873716), the National Key Research and Development Program of China (no. 2017YFA0104604), the Construction Engineering Special Fund of “Taishan Scholars” of Shandong Province (no. ts20190975 and tsqn201909180), the National Key R&D Program of China (no. 2017YFB0405400), the Key Research and Development Program of Shandong Province (no. 2018GSF118065), the Fundamental Research Funds of Shandong University (no. 2018JC005), the Collaborative Innovation Center of Technology and Equipment for Biological Diagnosis and Therapy in Universities of Shandong, and the Open Foundation of Shandong Provincial Key Laboratory of Oral Tissue Regeneration (no. SDKQ201901 and SDKQ201904).

## Conflict of Interest

The authors declare that the research was conducted in the absence of any commercial or financial relationships that could be construed as a potential conflict of interest.

## Supplementary Material

Supplementary Figure 1 ROC curve analysis of 1-, 3-, and 5-year OS in **(A)** HCC and **(B)** LUAD.
