# Supplementary figures and images for: AUNIP Expression Is Correlated With Immune Infiltration and Is a Candidate Diagnostic and Prognostic Biomarker for Hepatocellular Carcinoma and Lung Adenocarcinoma
Source: Front Oncol. 2020 Dec 9;10:590006. doi: 10.3389/fonc.2020.590006 (PMC7756081; doi:10.3389/fonc.2020.590006)

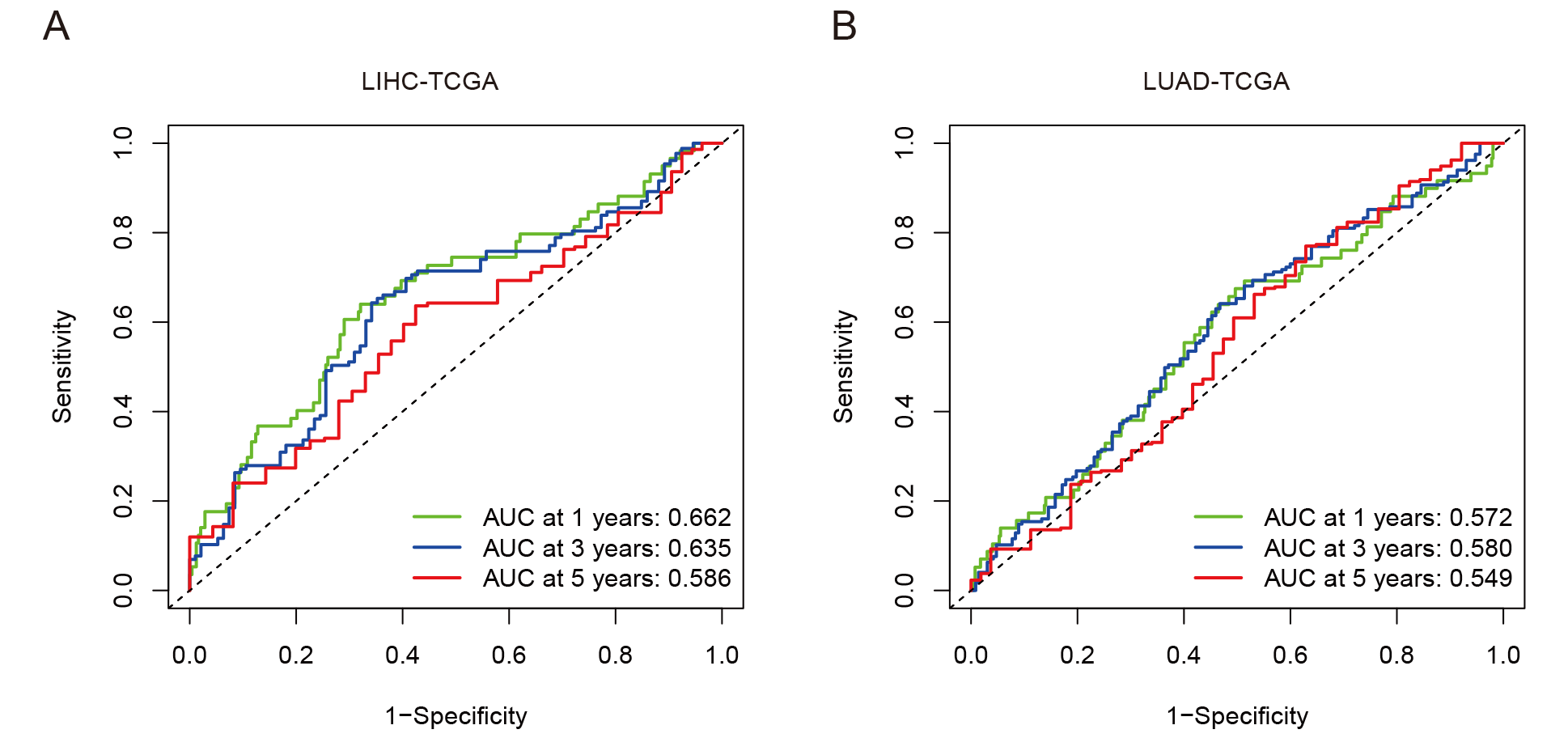

Supplement: Supplementary Figure 1 — ROC curve analysis of 1-, 3-, and 5-year OS in (A) HCC and (B) LUAD. [file Image_1.tif]
